# Traumatic ulcerative granuloma with stromal eosinophilia (TUGSE) in children: a case report and literature review

**DOI:** 10.1515/biol-2025-1328

**Published:** 2026-06-26

**Authors:** Haihong Wang, Juanjuan Zong

**Affiliations:** Affiliated Stomatological Hospital of Nanchang University, 330000, Nanchang, Jiangxi, China

**Keywords:** oral eosinophilic ulcer, children, TUGSE

## Abstract

Traumatic ulcerative granuloma with stromal eosinophilia (TUGSE) is a rare, self-limiting, chronic benign lesion of the oral mucosa, whose pathophysiology remains unclear. This article reports an 8-year-old male child who presented with recurrent oral ulcers for more than 2 years, with non-fixed ulcer locations. In the past year, the frequency of ulcer occurrences increased, and healing was delayed. In the past month, the pain intensified, affecting his eating. Oral examination revealed a 30 mm × 15 mm deep ulcer in the left lower posterior teeth area, covered with a yellow-white pseudomembrane and without surrounding hyperemia. Histopathology showed dense eosinophil infiltration and skeletal muscle inflammation. Immunohistochemistry demonstrated positivity for CD30, CD34 and other markers, confirming the diagnosis of TUGSE Treatment included correcting poor oral habits, topical application of growth factor gel, and symptomatic medication. Follow-up showed improvement in ulcer recurrence frequency and size, but complete control was not achieved. TUGSE in children manifests as refractory recurrences, requiring histopathological diagnosis to exclude malignancies and other diseases. Local treatment can alleviate symptoms, but long-term recurrences suggest the need for further exploration of underlying etiologies and personalized interventions.

## 1 Introduction

Traumatic ulcerative granuloma with stromal eosinophilia (TUGSE) is a rare, benign, and self-limiting condition whose etiology and pathophysiology remain incompletely understood [1], 2]. Clinically, it manifests as a well-demarcated ulceration with indurated margins, often leading to misdiagnosis due to its resemblance to malignant tumors or chronic infectious ulcers [1], 2]. Histologically, it is characterized with ulcerative mucosa, with diffuse inflammatory infiltration abundant in eosinophils, different numbers of T lymphocytes, and possibly monocytes, displaying dense, diffuse and deep infiltration, extending to submucosa and involving the underlying soft tissues, muscle fibers and salivary glands. [1], 2]. Although reported in both adults and children, the clinical characteristics and management strategies of pediatric cases, particularly those with recurrent or refractory manifestations, deserve further exploration [3]. Current treatment approaches primarily focus on local interventions, including glucocorticoids, growth factor gels, and symptomatic therapies [4], 5]. However, achieving long-term control of recurrences remains a clinical challenge. It has been reported that while local treatments may alleviate symptoms, they do not target potential underlying causes such as immune abnormalities or chronic trauma, which may contribute to recurrent episodes in some cases [5], 6]. Additionally, variations in treatment compliance among pediatric patients may further influence outcomes, indicating a need for personalized intervention strategies [7]. In this case report, we presented a recurrent TUGSE in an 8-year-old boy, characterized by shifting lesion locations, a 2-year disease duration, and recent exacerbation, offering a unique perspective on the diagnosis and management of atypical TUGSE in children.

## 2 Case information

### 2.1 General information

An 8-year-old male child presented with recurrent oral ulcers for more than 2 years, with indefinite locations. Over the past year, the ulcer episodes had become more frequent, healing cycles were prolonged, and the pain was pronounced, affecting his eating. Two weeks ago, an ulcer recurred on the right upper posterior gingival region. He was diagnosed with “recurrent aphthous ulcer” and was subjected to treatment with recombinant bovine basic fibroblast growth factor gel, compound pearl oral ulcer granules, licorice zinc granules, Xibei gingival fluid, and hormonal gargle. Following treatment, the ulcer on the right upper posterior tooth healed almost completely; however, an ulcer on the left lower posterior tooth recurred. The patient denied having eye and genital ulcers, systemic diseases, a family history, or a history of drug allergies.

**Figure 1:**
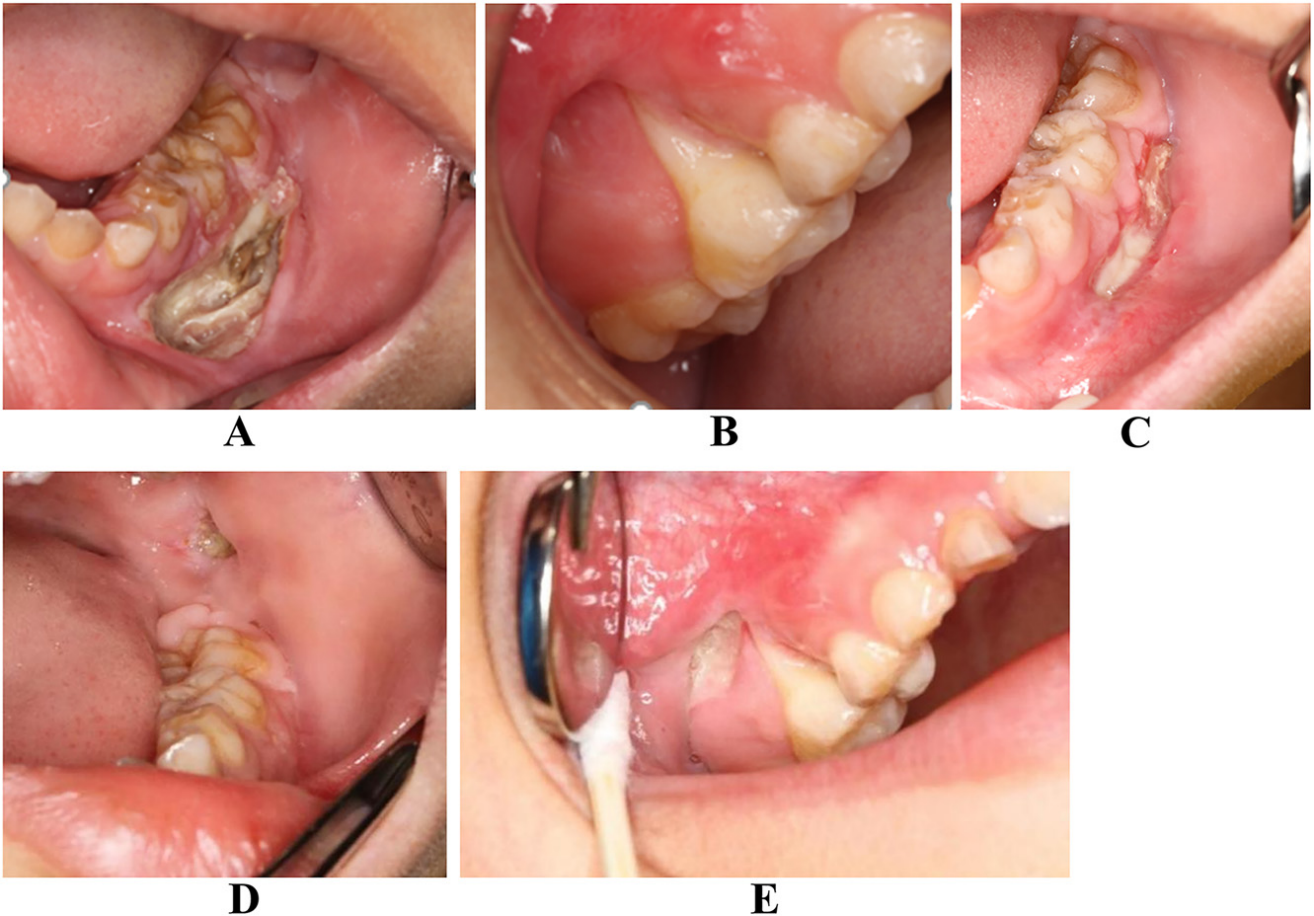
Intraoral photographs. (A) Initial diagnosis; (B) initial diagnosis; (C) 4 days after biopsy; (D) 1 week after biopsy; (E) recurrence of gingival ulcer on the right upper posterior tooth.

Intraoral examination revealed a large crater-like ulcer at the mucosal fold of gingiva, measuring approximately 30 mm × 15 mm. The ulcer was covered with a yellow-white pseudomembrane, had a clear peripheral boundary, and the surrounding mucosa showed no significant congestion or redness (Figure 1A). Additionally, the buccal gingiva demonstrated evident recession, and the ulcer was in the process of healing (Figure 1B). During the consultation, it was observed that the child had a habit of constantly moving their mouth and lips. Cone Beam Computed Tomography (CBCT) examination of the jaws revealed no abnormalities, and blood tests were also unremarkable. Bacterial culture and identification of the exudate from the lesion surface yielded no significant findings.

**Figure 2:**
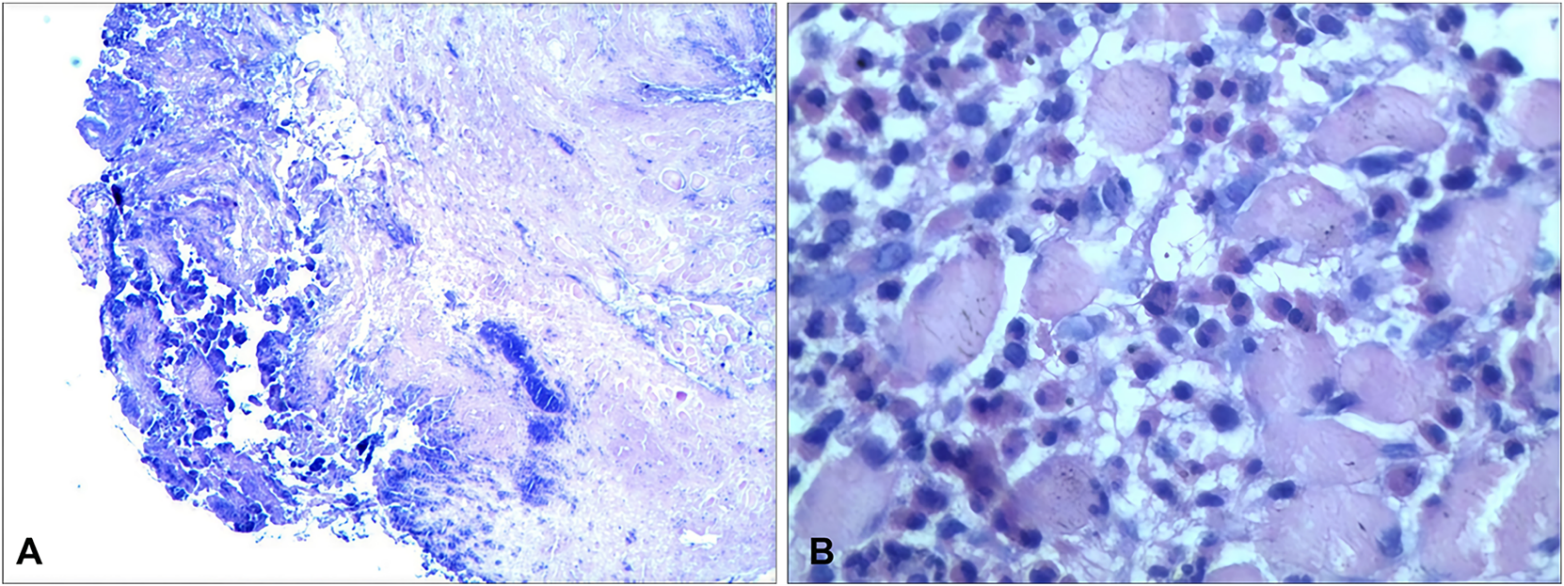
HE staining of the lesion in the left lower posterior region surface fibrinous exudate, bacterial colonies and necrotic tissue overlay, dense submucosal infiltration of neutrophils, eosinophils and lymphomonocytes, inflammatory infiltration into skeletal muscle. (A) ×10 magnification; (B) ×40 magnification.

**Figure 3:**
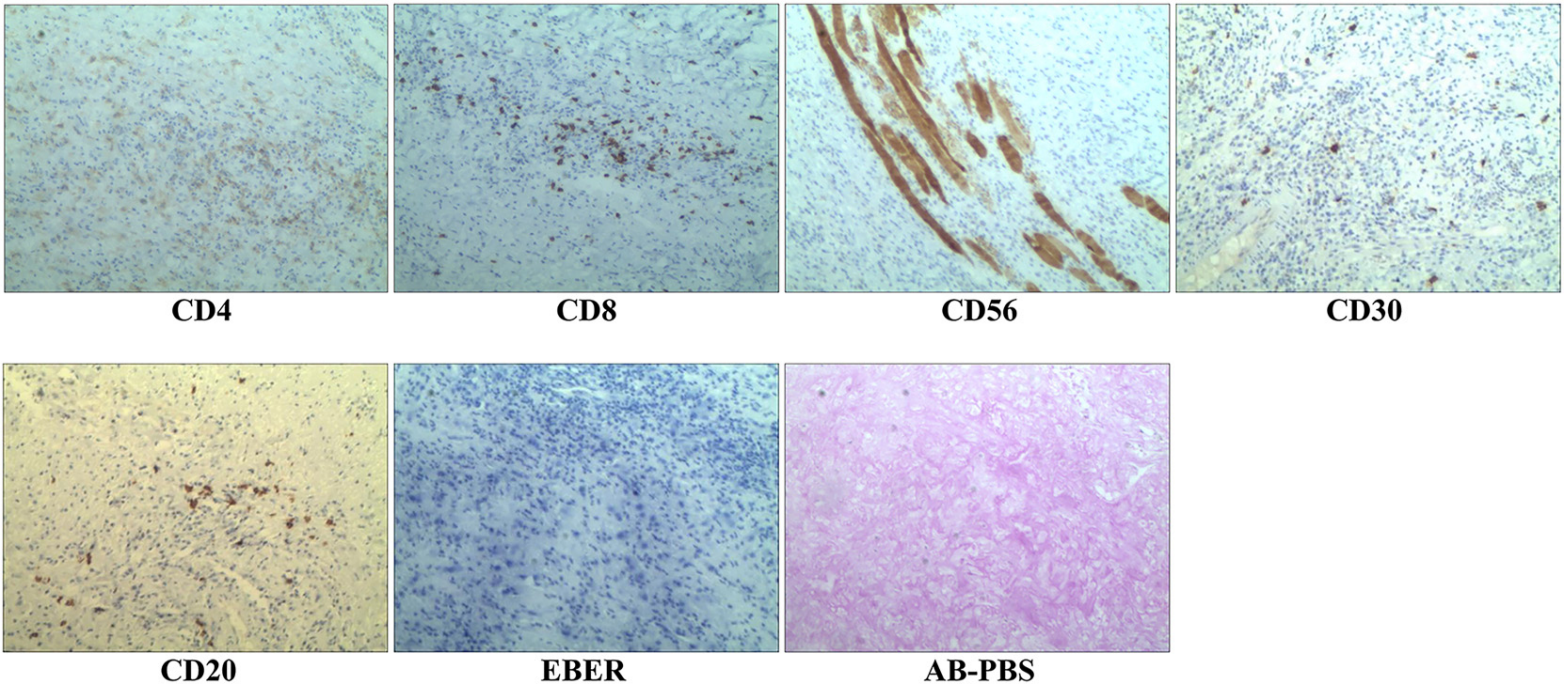
Immunohistochemical staining for CD4, CD8, CD56, CD30, EBER, AB-PBS (×10). Immunohistochemistry of the lesion area was diffusely positive for CD4, CD8 and CD30, while negative for EBER and PAS.

Under local anesthesia, a biopsy of the left posterior mandibular region was performed. HE staining showed dense infiltration of inflammatory cells and eosinophils, with fibrous exudate formation on the surface and inflammatory infiltration and lymphocyte infiltration in the deep skeletal muscle (Figure 2A and B). Immunohistochemistry of the lesion area was diffusely positive for CD4, CD8 and CD30, while negative for EBER and PAS (Figure 3A–E). The child was diagnosed with TUGSE.

Local symptomatic treatment for the ulcer included the use of a novel mouthwash for rehabilitation, application of Obrocaine gel to mitigate discomfort, and application of recombinant bovine basic fibroblast growth factor gel. The family was instructed to oversee the correction of the child’s habit of constantly moving his lips and mouth. One week after the histopathological biopsy, the ulcer had healed almost completely (Figure 1C and D). However, there was a recurrence of ulcers on the left cheek mucosa and right posterior maxillary gingiva. The ulcer on the left cheek mucosa measured approximately 5 mm × 5 mm, while the ulcer on the right posterior maxillary gingiva measured approximately 10 mm × 5 mm. Both ulcers were crater-like, with clear peripheral boundaries, covered with a yellow-white pseudomembrane, and the surrounding mucosa showed no significant congestion or redness (Figure 1E).

Currently, telephone follow-up has revealed that the child has ceased the habit of moving their mouth and lips. Although the child still experiences recurrences of oral mucosal ulcers, the frequency and size of ulcer outbreaks have significantly improved, and there are no other systemic symptoms.

**Informed consent:** Informed consent has been obtained from all individuals included in this study.

**Ethical approval:** The research related to human use has been complied with all the relevant national regulations, institutional policies and in accordance with the tenets of the Helsinki Declaration, and has been approved by the Medical Ethics Committee of the Affiliated Stomatological Hospital of Nanchang University (Approval No. 2026(032)).

## 3 Discussion

TUGSE is a rare, chronic, self-limiting benign ulcerative lesion of the oral mucosa. In 1956, Popoff first reported a lesion similar to TUGSE in adults, which he believed to be a variant of facial sarcoidosis [8]. Later in 1970, Shapiro described it as a distinct disease entity of the tongue mucosa [9]. Based on a literature review in 1983, Elzay linked the commonly reported Riga-Fede disease in infants with oral ulcers in adults, noting their clinical and histopathological similarities [10]. Consequently, EUOM was often previously referred to as Riga-Fede disease [11]. Subsequently, various terms emerged to describe this condition, such as TUGSE, oral traumatic granuloma, and eosinophilic ulcer of the tongue [12], [13], [14]. In 1997, Ficarra and colleagues proposed that EUOM might represent the oral manifestation of cutaneous CD30 lymphoproliferative disorders (LPD30) based on clinical, histological, and antigenic features [15], or at least some cases of EUOM might represent intraoral LPD30 [2], [16], [17], [18]. Reports of lymphomatoid papulosis (LyP), a subclass of LPD30, are particularly frequent [19]. Oral LyP is considered a secondary extra-cutaneous manifestation of cutaneous CD30 LPD, although up to half of the reported cases show only oral EUOM without cutaneous manifestations [20]. Studies have shown that TUGSE partially represents CD30-positive LPD, and its histopathology is similar to LyP. Oral LyP is rare, primarily type A/C. Approximately one third of patients have skin involvement before the incidence of oral lesions. In children, LyP is often accompanied by skin involvement, and mucosal surface damage is even more rare. The pathological feature of LyPA is that CD4+/CD30+Reed-Sternberg-like cells are evenly distributed in other inflammatory conditions, and compact flaky infiltrating CD4+/CD30+pleomorphic cells in the dermis/lamina propria-submucosa of LyPC [21]. According to the clinical characteristics of rapid healing after biopsy and the pathological characteristics of LyPA/C, this case is classified as TUGSE.

The etiology and pathophysiology of TUGSE remain unclear, although most agree that TUGSE is closely associated with traumatic factors [10]. Scholars believe that persistent trauma may lead to differences in tissue antigens or introduce toxins, microorganisms, endogenous breakdown products, or exogenous proteins into the tissue [22]. Additionally, trauma itself can trigger a local immune response [23]. It has also been reported that TUGSE is mediated by a T-cell immune response; toxic substances released by eosinophils, increased mast cells, or cytokines and chemokines may play a significant role in its development [12], 14]. Recently, several cases have suggested a possible link between TUGSE and EBV infection. Abdel-Naser [24] was the first to describe the potential role of EBV infection in the pathophysiology of TUGSE. Hong et al. [16] found that two pediatric patients with LPD30 clinically presenting as EUOM tested positive for EBV, leading the researchers to conclude that pediatric TUGSE or LPD30 is a manifestation of EBV-positive LPD30, with a pathophysiology and etiology distinct from that of adults. However, there are few reports on the EBV status of pediatric and adult TUGSE patients, mostly limited to case reports, necessitating further research to provide more compelling data. In this particular case, the child tested negative for EBV and positive for CD30. To date, the etiology and pathophysiology of TUGSE remain elusive. Frequent recurrence may be related to long-term duration of oral ulcer, causing the child to have bad oral habits of involuntary lip and tongue movements, or even putting fingers into mouth and leading to local trauma. Subsequently, oxybupivacaine gel was locally given to alleviate the discomfort caused by ulcer, helping the child gradually get rid of bad habitual lip.

The clinical manifestations of TUGSE include rapidly developing ulcers, which may be painful or painless, with a white to yellow base, hardened and elevated edges, and a clear perimeter. These ulcers can occur on any mucosal surface, most commonly on the tongue, followed by the cheek mucosa, mucosal folds, lips, gingiva, and retromolar areas. Multiple ulcers may appear simultaneously or recur, persisting for several weeks or longer, and exhibit a self-limiting nature. Biopsy can accelerate the disease’s self-healing properties [9], 13], 14]. The pathological features of TUGSE are characterized by an abundance of eosinophils. Studies have suggested that a large number of eosinophils cannot produce sufficient transforming growth factors to attenuate the wound healing process. Biopsy stimulates high expression of transforming growth factors, activating a process similar to normal wound healing, thereby leading to rapid healing [25]. The age of onset for TUGSE follows a bimodal distribution, with the first peak occurring in early childhood and the second peak in older adults around 60 years of age. Males appear to be affected more frequently than females (1.6:1) [25]. The typical pathological features of TUGSE include diffuse, polymorphous inflammatory infiltration extending into the submucosa and involving the underlying soft tissue, skeletal muscle layer, and salivary glands, which can lead to degeneration of the underlying muscles. The infiltrate consists primarily of eosinophils and varying numbers of T lymphocytes and mast cells. Large, atypical, and mitotically active mononuclear cells may be present, and there may be a significant increase in the number of blood vessels [2], 10], [26], [27], [28].

Due to the similarity in clinical and histological manifestations, the differential diagnosis of TUGSE should include syphilis, squamous cell carcinoma, other malignancies (such as leukemic infiltration), metastatic carcinoma and lymphoma of the oral mucosa, eosinophilic vasculitis-like proliferative lesion, Langerhans cell histiocytosis, chronic granulomatous diseases (tuberculosis, Wegener’s granulomatosis, cutaneous tuberculosis), pyogenic granuloma, viral infections (herpes simplex virus), and mucosal manifestations of systemic diseases [8], 9], 14], 25], 27], 29]. A detailed medical history and typical clinical manifestations often aid in the initial determination of etiology, and in most cases, a biopsy is recommended to rule out malignancies. If there is a clear traumatic stimulus, TUGSE should be eliminated first. Reported treatment methods include local glucocorticoid injection, gargling, topical antibiotics, curettage, and cryotherapy. The most commonly used treatment is simple surgical removal [25]. TUGSE has a good prognosis with a low recurrence rate. Schwartz et al. [2] reported a history of recurrent oral ulcers in 5 patients with TUGSE after treatment, but other symptoms had disappeared, and long-term follow-up was recommended.

## 4 Conclusions

TUGSE is a rare benign, chronic, self-limiting ulcerative lesion of the oral mucosa. Its etiology and pathophysiology remain unclear. Most case reports indicate the presence of EBV positivity in both TUGSE patients and LPD30 patients presenting as TUGSE, suggesting that EBV may play a role in the pathophysiology of TUGSE. However, further support from future studies is still needed. Additionally, the use of multiple terms in the literature to describe TUGSE hinders our understanding of the condition. Therefore, the standardization of terminology will also aid in our investigation of its etiology and pathophysiology.
